# Inequality of opportunity in outpatient expenditure among the elderly with multimorbidity: evidence from China

**DOI:** 10.1186/s12939-023-01953-z

**Published:** 2023-08-14

**Authors:** Kangkang Zhang, Hua You, Linxiang Yu, Qifeng Wu, Xinpeng Xu

**Affiliations:** 1 School of Health Policy & Management, Nanjing Medical University, Nanjing, China; 2 School of Public Health, Nanjing Medical University, Nanjing, China; 3 Institute of Healthy Jiangsu Development, Nanjing Medical University, Nanjing, China; 4 Department of Cardiothoracic Surgery, Jiangsu Province Hospital of Chinese Medicine, Nanjing, China

**Keywords:** Multimorbid elderly, Outpatient expenditure, Inequality of opportunity, Parametric approach, Outpatient reimbursement, China

## Abstract

**Background:**

Inequality of opportunity (IOp) stemming from social circumstances exists in outpatient service utilization for the multimorbid elderly in China. However, little is known regarding the magnitude of the IOp and its composition. Therefore, this study aims to measure the IOp in outpatient expenditure and provide potential pathways for policy reform by assessing the contribution of each circumstance.

**Methods:**

This study included 3527 elderly aged ≥ 65 years with multimorbidity from the Chinese Longitudinal Healthy Longevity Study conducted in 2017–2018. An ordinary least squares regression model was used to analyze the circumstance-influencing factors of outpatient expenditure. The parametric approach was performed to quantify the IOp in outpatient expenditure and the Shapley value decomposition method was employed to determine the contribution of each circumstance. By extracting heterogeneity in the residual of the circumstance-dependent equation of outpatient expenditure across circumstance groups divided based on cluster analysis, we captured the effect of unobserved circumstances.

**Results:**

Except for pension and distance to health facilities, all the associations between circumstance and outpatient expenditure were statistically significant. The inequality caused by circumstances accounted for 25.18% of the total inequality. The decomposition results revealed that the reimbursement rate contributed 82.92% of the IOp, followed by education duration (4.55%), household registration (3.21%), household income (3.18%), pension (1.49%), medical insurance (1.26%), physical labor (0.99%), unobserved circumstances (0.86%), distance to health facilities (0.83%) and region (0.71%).

**Conclusions:**

The priority of policy enhancement is to effectively improve the outpatient reimbursement benefit for treating chronic diseases. Additional crucial actions include enhancing the health literacy of the multimorbid elderly to promote the shift from medical needs to demands and accelerating the construction of rural capacity for providing high-quality healthcare to the elderly with multimorbidity.

**Supplementary Information:**

The online version contains supplementary material available at 10.1186/s12939-023-01953-z.

## Background

Multimorbidity (defined as the coexistence of ≥ 2 chronic conditions) is becoming increasingly prevalent among the elderly, and its prevalence grows substantially with age [1]. A recent integrative literature review found that the prevalence of multimorbidity among the elderly aged ≥ 60 years was between 30.7% and 57% [2]. Furthermore, globally or in China, the challenge of population aging will intensify. There is no doubt that the scale of the elderly population with multimorbidity will keep expanding, resulting in enormous medical needs. Polypharmacy, which usually refers to the daily use of five or more medications, is common among the elderly patients, particularly those with multimorbidity, for whom one or more medications may be used to treat each condition [3]. In Shanghai, the prevalence of polypharmacy was 68.6% among the elderly aged ≥ 65 years [4]. Hospital outpatient clinics are a common channel for purchasing and consulting on medication for patients with chronic diseases [5], and evidence showed that the number of outpatient visits of patients to medical institutions within two weeks increased by an average of 44% for each additional chronic disease [6]. Meanwhile, the majority of the increase in outpatient expenditure has been attributable to an increase in outpatient visits [7]. Therefore, for the elderly with multimorbidity, outpatient expenditure is an important indicator of outpatient service utilization in the treatment of chronic diseases. However, healthcare expenditure, including outpatient expenditure, is frequently related to multiple social factors, causing gross inequalities that impede the elderly with multimorbidity from realizing their full health potential.

Among all the factors influencing outpatient expenditure, the first type is known as socioeconomic status, such as income [8], occupation [9], and education [10]. The second type of influencing factor stems from the individual’s residence location, including country [11], urban-rural [12], and region [13]. The third type is the healthcare accessibility factor, which represents the affordability and convenience of healthcare, e.g., medical insurance [14], outpatient reimbursement [15], and distance to health facilities [16]. The fourth type of influencing factor is medical needs. Many researchers have emphasized the role of disease and its severity in rising outpatient expenditure [17, 18]. In addition, some studies focused on the association between outpatient expenditure and gender, as well as age [19]. However, not every source of the outpatient expenditure inequality is unfair. Accurate identification of unreasonable components of inequalities in outpatient expenditure among the elderly with multimorbidity is essential to the formulation and improvement of health policy.

The inequality of opportunity (IOp) provides a feasible framework for evaluating the fairness of the factors affecting outpatient expenditure. The concept of IOp first appeared in Rawls’ work [20]. On this premise, Roemer introduced the philosophical connotation of IOp into economics in the mathematics form [21–23]. He believes that one’s outcome, namely, personal welfare in terms of living, education, and healthcare, is determined by two categories of variables: circumstances (initially called “types”) and efforts; the former is out of one’s control, while the latter is not. The inequality of outcome resulting from circumstances is unfair, whereas resulting from efforts is fair. Notably, Roemer emphasizes the partial effect of circumstance, that is, one’s effort is greatly influenced by circumstance. In other words, IOp consists of two portions, one generated directly by circumstance and the other mediated by effort, which all requires the government to take responsibility.

Considering the two main principles, namely vertical equity (people with greater medical needs should receive more healthcare than those with lesser needs) and horizontal equity (equal treatment for equivalent needs) in classical health economics [24], it is completely practicable to equate medical needs with effort. This is because inequalities in outpatient expenditure resulting from medical needs are equity, and numerous studies have reported that people in different socially determined settings have different medical needs [25–27], i.e., the medical needs are subject to the partial effect of circumstances, as with effort in the framework of IOp. Consequently, among the influencing factors reviewed above, socioeconomic status, residence location, and healthcare accessibility can be categorized as circumstances, while medical needs can be categorized as effort. Gender and age have both circumstance and effort attributes [28], their processing in the analysis of the IOp in outpatient expenditure for the multimorbid elderly will be detailed in the Methods section.

Currently, researchers have primarily explored the IOp in health status [29–31]. Although equitable access to healthcare is an integral part of achieving health equity [32], little attention has been paid to the IOp in healthcare expenditure and no study on the elderly population with multimorbidity has been found. For the measurement of IOp, there are ex-ante (dividing individuals under the same circumstances into the same group and defining inequality between groups as IOp) and ex-post (dividing groups according to the differences in effort levels and defining inequality within the group as IOp) methods [33]. Since objectively measuring the level of effort is difficult, the ex-ante approach is more widely applied. But a limitation of the ex-ante approach is that it provides only the lower-bound estimates of IOp. This is mainly due to the fact that the portion of inequality owing to unobserved circumstances might be incorrectly attributed to effort and luck rather than the IOp [34]. This study focuses on the elderly population with multimorbidity in China, aims to measure the IOp in outpatient expenditure closer to its actual value using an ex-ante approach under the consideration of unobserved circumstances, and proposes prior and targeted policy improvement countermeasures based on the contribution of each circumstance factor to the IOp.

## Methods

### Data source

Data used in our study were from the latest 8th wave of the Chinese Longitudinal Healthy Longevity Study (CLHLS) conducted by the Center for Healthy Aging and Development of the National School of Development at Peking University in 2017–2018, which randomly selected approximately half of the counties and city districts of 23 Chinese provinces, whose populations together constitute approximately 85% of the total in China. This dataset contains basic individual and household information, emotional characteristics, behavioral lifestyle, health status, and healthcare expenditure for 15,498 elderly aged ≥ 65 years. A detailed description of the sampling design and data quality was reported elsewhere [35].

The CLHLS classified chronic diseases into 25 categories: hypertension, diabetes, heart disease, stroke/cerebrovascular disease, bronchitis/emphysema/asthma/pneumonia, pulmonary tuberculosis, cataracts, glaucoma, cancer, prostate tumor, gastric or duodenal ulcer, Parkinson’s disease, bedsore, arthritis, dementia, epilepsy, cholecystitis/cholelithiasis, dyslipidemia, rheumatism or rheumatoid disease, chronic nephritis, breast disease, uterine fibroids, prostatic hyperplasia, hepatitis, and others, which allowed us to analyze the IOp among those elderly with multimorbidity. According to the definition, there were 5163 multimorbid elderly in this dataset. After excluding those with missing values or outliers for the selected variables, a final sample of 3527 valid observations (68.3%) was utilized in the analysis.

### Variable selection

#### Outcome variable

Referring to the general practice of setting outcome variables in prior studies of healthcare inequality [36], this study used the outpatient expenditure of the multimorbid elderly in the previous year (including out-of-pocket expenses and the reimbursement component) as the outcome variable.

#### Circumstance variables

As mentioned in the Background section, circumstance variables falling under socioeconomic status, residence location, and healthcare accessibility were selected from the CLHLS dataset. Socioeconomic status included annual household income (How much was the total income of your family last year? CNY), physical labor (Did you often do physical labor in the past? 0 = no; 1 = yes), pension (Do you have an old-age pension or a pension for retirement? 0 = no; 1 = yes), and education duration (How many years of education have you received?). Residence location included household registration (0 = urban; 1 = rural) and region (division criteria from the National Development and Reform Commission, 0 = eastern; 1 = central and western). Healthcare accessibility included medical insurance (according to the level of protection: 1 = none; 2 = New Rural Cooperative Medical Scheme, NRCMS, low-level; 3 = Urban Resident Basic Medical Insurance/Urban Employee Basic Medical Insurance, URBMI/UEBMI, middle-level; 4 = commercial medical insurance/free medical treatment, CMI/FMT, high-level) [37], reimbursement rate (the proportion of reimbursement component to outpatient expenditure) and distance to health facilities (How far is it from your home to the nearest hospital? km).

Gender is routinely regarded as a circumstance variable in the studies of economic income and educational opportunities due to its close relationship to the external circumstances in which individuals live [38, 39]. However, the spectrum of chronic diseases in the elderly varies by gender, as evidenced by the distinctness in specific chronic conditions (e.g., uterine fibroids and prostatic hyperplasia) and the different prevalence of the same diseases, which results in the differences in their needs for healthcare. In accordance with the principle of vertical equity, the inequality in outpatient expenditure caused by medical needs is fair. Indeed, there is a similar situation with the age variable. Therefore, instead of directly incorporating age and gender into the category of circumstances, this study considered the portions associated with selected circumstances of age, gender, and other factors that cannot be clearly distinguished (see next subsection). In addition, the variables age, gender, and number of chronic diseases were selected to describe the elderly with multimorbidity.

### Statistical analysis

According to the parametric approach [40, 41], the determining equation of outpatient expenditure $y$ for individual $i$ was developed:

1 $${lny}_{i}=\alpha +\beta {C}_{i}+\gamma {E}_{i}+{\mu }_{i}$$

where ${C}_{i}$ denotes circumstance variables, ${E}_{i}$ denotes effort variables, $\alpha$ is a constant, and ${\mu }_{i}$ is the residual term. To eliminate bias due to large variances, we adopted the natural logarithm of outpatient expenditures and household incomes. Since ${C}_{i}$ is exogenous, the ${E}_{i}$ can be expressed as a linear function of the circumstance variables:

2 $${E}_{i}^{l}={\eta }^{l}+{\rho }^{l}{C}_{i}+{e}_{i}^{l}$$

where ${E}_{i}^{l}$ is the $l$th effort variable. Eq. (2) was then substituted into Eq. (1):

3 $${lny}_{i}=\lambda +\tau {C}_{i}+{\epsilon }_{i}$$

where the coefficient $\tau$ contains two components: first, the direct effect of circumstances on outpatient expenditure; and second, the indirect effect via efforts. Using the estimated values of $\lambda$ and $\tau$, along with the true values of circumstance variables, a smoothed distribution of outpatient expenditure {${ln\widehat{y}}_{i}$} was obtained, i.e., ${\widehat{y}}_{i}=\text{e}\text{x}\text{p}(\widehat{\lambda }+\widehat{\tau }{C}_{i})$. The absolute (IOA) and relative (IOR) amount of IOp can be defined as follows [34]:

4 $$IOA=I\left(\right\{{\widehat{y}}_{i}\left\}\right)$$

5 $$IOR=I\left(\right\{{\widehat{y}}_{i}\left\}\right)/I\left(\right\{{y}_{i}\left\}\right)$$

where $\text{I}(\cdot )$ is a measure of inequality based on the generalized entropy index $\text{G}\text{E}\left(0\right)$.

fSince Eq. (3) cannot be exhaustive of all circumstance factors, it always misses some difficult to observe. Thus, ${\epsilon }_{i}$ comprises two parts, one is related to known circumstances but undefined, which can be called unobserved circumstances (UC), while the other accounts for the net effort and sheer luck whose expectation is zero after removing the effect of circumstances. Individuals in the same known circumstances were classified into the same group. The distribution of the part affected by known circumstances is homogeneous within the same group and heterogeneous across groups; the distribution of the other part not affected by known circumstances is completely random and homogeneous both within and between groups. The heterogeneity of unobserved circumstances between groups leads to heteroskedasticity in the residual term ${\epsilon }_{i}$, with each group $n$ having a different variance ${\sigma }_{n}^{2}=Var\left[{\epsilon }^{n}\right|{C}^{n}]$. We divided the residual term ${\epsilon }_{i}$ in Eq. (3) for identifying the unobserved circumstances [42], and the steps were as follows.

(I) All circumstance variables were normalized and the k-medians algorithm was applied to cluster analysis. The optimal number of clusters was determined to be 6 within the range of 3–15 based on the Calinski-Harabasz pseudo-F index, and individuals within the same cluster were considered to have the same external circumstances. Denoting the circumstance groups as $m$ ($m$=1, 2, …, 6), the outpatient expenditure in each group was independently estimated:
  6 $$ln{y}_{i}^{m}=\lambda^{m} +\tau {C}_{i}^{m}+{\epsilon }_{i}^{m}$$
  where ${C}_{i}^{m}$ is the vector of circumstances in group $m$ and ${\epsilon }_{i}^{m}$ is the corresponding residual term. At this point, ${\epsilon }_{i}^{m}$ within the same group is homogeneous. Based on the estimation results of Eq. (6), the variance estimator ${\widehat{\sigma }}_{m}^{2}$ of the residual term within each group was obtained. Using the proportion ${f}_{m}$ of the number of samples in each group to total samples, the overall variance ${\sigma }^{2}=\sum _{m=1}^{6}{f}_{m}{\widehat{\sigma }}_{m}^{2}$ was calculated by weighted summation of the variances of all groups. Then, the weight index $k=1/\sigma$ was calculated based on the overall variance ${\sigma }^{2}$.
(II) The heterogeneity between groups was extracted using the weight index:
  7 $$ln{y}_{i}=\lambda +\tau {C}_{i}+({\epsilon }_{i}-{\epsilon }_{i}/k{\widehat{\sigma }}_{m})+{\epsilon }_{i}/k{\widehat{\sigma }}_{m}$$
  where $({\epsilon }_{i}-{\epsilon }_{i}/k{\widehat{\sigma }}_{m})$ can be denoted as ${\theta }_{i}$ and ${\epsilon }_{i}/k{\widehat{\sigma }}_{m}$ as ${\upsilon }_{i}$. Therefore, ${\theta }_{i}$ is the heteroskedasticity component across circumstance groups after excluding the within-group homoskedasticity, which represents unobserved circumstances. ${\upsilon }_{i}$ is the homoskedasticity component with an expected value of zero and a distribution that is identical across groups. We recalculated the IOA and IOR according to Eq. (7), which means that the contribution of unobserved circumstances was included. Additionally, the Shapley value decomposition approach [43] was utilized to decompose the IOR for determining the contribution of each circumstance factor in creating disparities in outpatient expenditure. This approach has the advantages of being independent of the ranking of circumstance variables and calculating the total IOp by adding the contributions of all circumstances [42].

The mean (standard deviation) and frequency (percentage) were used to describe numerical and categorical variables, respectively. In accordance with Eq. (3), the ordinary least squares (OLS) regression of circumstance factors influencing outpatient expenditure was performed first. The robust standard error was utilized and all *P* values were two-tailed. All statistical analyses were conducted using Stata V.17.0 (Stata Corp).

To attenuate the inverse causality between actual outpatient reimbursement rates and outpatient expenditure due to the large medical expense reimbursement policy, i.e., higher expenses may lead to an increase in reimbursement rates, we multiplied the reimbursement rates by 0.9 as a penalty for the participants with outpatient expenditure over 10,000 CNY (a lower threshold for large medical expense reimbursement policies [44]), with outpatient reimbursement rates over 85% (a higher outpatient reimbursement rate [45]) and enrolled in NRCMS, URBMI, and UEBMI. Then we repeated the above analysis and obtained very close results (see Additional file 1), indicating that the reverse causality caused by large medical expense reimbursement policies contributes little to the IOp.

### Robustness analysis

As the different clusters in cluster analysis affect the extraction of unobserved circumstances, this study set the number of clusters to 5 and 7, respectively, then recalculated and re-decomposed the IOR in outpatient expenditure among the elderly with multimorbidity to verify the robustness of results. In addition, to evaluate the selection bias resulting from sample exclusion, sensitivity analysis was conducted using the same methods with 20 complete datasets imputed by the Markov Chain Monte Carlo (MCMC) algorithms [46]. And we specified the number of clusters as 10 to facilitate results combining.

### Ethics statement

This is a secondary analysis using the CLHLS data, and the original study was approved by the Research Ethics Committees of Duke University and Peking University (IRB00001052–13074).

## Results

### Characteristics of the study population

The average age of the 3527 multimorbid elderly was 83.60 ± 10.91 years. They spent an average of 665.97 ± 1467.22 USD in outpatient services in the previous year while suffering from 3.25 ± 1.74 chronic conditions. Their average distance to the nearest health facilities was 2.36 ± 5.46 km; the average reimbursement rate for outpatient expenditure was 0.29 ± 0.38; the average annual household income was 7431.41 ± 5663.43 USD; and the average education duration was 4.43 ± 4.90 years. There were 2403 (68.13%) elderly who used to do physical labor regularly; 1222 (34.65%) had no pension; and 401 (11.37%) had no medical insurance. Table 1 also shows that the proportion of female elderly was 55.68% (1964); the proportion of rural elderly was 56.34% (1987); and the proportion of central and western elderly was 41.76% (1473).

**Table 1:** Characteristics of the study population (N = 3527)

Variables	Mean/N	SD/%
Outpatient expenditure (USD)	665.97	1467.22
Age (year)	83.60	10.91
Number of chronic diseases	3.25	1.74
Household income (USD)	7431.41	5663.43
Education duration (year)	4.43	4.90
Reimbursement rate	0.29	0.38
Distance to health facilities (km)	2.36	5.46
Age group		
65–74	879	24.92
75–84	1037	29.40
85–94	910	25.80
≥ 95	701	19.88
Gender		
Male	1563	44.32
Female	1964	55.68
Physical labor		
No	1124	31.87
Yes	2403	68.13
Pension		
No	1222	34.65
Yes	2305	65.35
Household registration		
Urban	1540	43.66
Rural	1987	56.34
Region		
Eastern	2054	58.24
Central and western	1473	41.76
Medical insurance		
None	401	11.37
NRCMS	1679	47.60
URBMI/UEBMI	1239	35.13
CMI/FMT	208	5.90

The annual average exchange rate of USD against CNY in 2017 was 6.7518 obtained from the National Bureau of Statistics

### OLS regression on outpatient expenditure

The results showed that regardless of whether the effect of unobserved circumstances was considered, higher household income (P < 0.01), frequent physical labor in the past (P < 0.01), living in central or western China (P < 0.1), longer education duration (P < 0.01), and higher outpatient reimbursement rates (P < 0.01) were significantly associated with higher outpatient expenditure while being a rural resident was the opposite (P < 0.1). Participating in NRCMS (P < 0.01), URBMI/UEBMI (P < 0.01), or CMI/FMT (P < 0.01) was associated with lower outpatient expenditure. The associations between outpatient expenditure and having a pension, distance to health facilities, and unobserved circumstances were not statistically significant (Table 2).

**Table 2:** Circumstance factors influencing outpatient expenditure

Circumstances	Outpatient expenditure (log)			
	(1)	(2)	(3)	(4)
Socioeconomic status				
Household income (log)	0.066***	0.067***	0.065***	0.067***
	(3.831)	(3.830)	(3.739)	(3.832)
Physical labor (ref: no)	0.290***	0.290***	0.296***	0.287***
	(4.359)	(4.367)	(4.412)	(4.246)
Pension (ref: no)	0.042	0.043	0.043	0.042
	(0.662)	(0.677)	(0.681)	(0.661)
Education duration	0.024***	0.024***	0.024***	0.024***
	(3.620)	(3.617)	(3.687)	(3.634)
Residence location				
Household registration (ref: urban)	–0.144*	–0.145*	–0.150*	–0.146*
	(–1.678)	(–1.683)	(–1.724)	(–1.696)
Region (ref: eastern)	0.105*	0.104*	0.101*	0.103*
	(1.864)	(1.845)	(1.799)	(1.803)
Healthcare accessibility				
NRCMS (ref: none)	–0.376***	–0.376***	–0.378***	–0.375***
	(–3.677)	(–3.675)	(–3.695)	(–3.667)
URBMI/UEBMI (ref: none)	–0.304***	–0.304***	–0.301***	–0.302***
	(–2.967)	(–2.959)	(–2.954)	(–2.950)
CMI/FMT (ref: none)	–0.547***	–0.546***	–0.546***	–0.544***
	(–3.779)	(–3.776)	(–3.787)	(–3.751)
Reimbursement rate	2.137***	2.134***	2.123***	2.132***
	(27.338)	(27.073)	(27.141)	(27.237)
Distance to health facilities	–0.008	–0.008	–0.008	–0.008
	(–1.607)	(–1.605)	(–1.578)	(–1.604)
Unobserved circumstances		0.048	0.875	0.075
		(0.145)	(0.956)	(0.286)
Constant	5.818***	5.818***	5.832***	5.821***
	(27.360)	(27.332)	(27.296)	(27.312)
Adjusted R^2^	0.247	0.247	0.247	0.247
Observations	3527	3527	3527	3527

The unobserved circumstances in columns (2), (3), and (4) were extracted based on the cluster analysis with 6, 5, and 7 clusters, respectively

The t-values calculated based on robust standard errors are reported in the parentheses

*** P < 0.01, ** P < 0.05, * P < 0.1

### Measurement and decomposition of IOp

The total inequality index $\text{G}\text{E}\left(0\right)$ of outpatient expenditure for the elderly with multimorbidity was 0.0341. After considering unobserved circumstances, the IOA was 0.0086 and the IOR was 0.2518. Namely, the inequality caused by circumstances accounted for 25.18% of the total. Table 3 presents the decomposition results of the IOR without and with the inclusion of unobserved circumstances. For the latter, the contribution of a single circumstance, reimbursement rate, achieved 82.92%, followed in descending order by education duration (4.55%), household registration (3.21%), household income (3.18%), pension (1.49%), medical insurance (1.26%), physical labor (0.99%), unobserved circumstances (0.86%), distance to health facilities (0.83%), and region (0.71%). From the circumstance categories, the contribution of socioeconomic status to the IOp in outpatient expenditure was 10.21%, residence location was 3.92%, and healthcare accessibility was 85.01%.

**Table 3:** Decomposition of the IOR in outpatient expenditure

Circumstances	Without UC		With UC	
	IOR	Contribution (%)	IOR	Contribution (%)
Socioeconomic status				
Household income	0.0079	3.14	0.0080	3.18
Physical labor	0.0025	0.98	0.0025	0.99
Pension	0.0037	1.45	0.0038	1.49
Education duration	0.0115	4.57	0.0115	4.55
Residence location				
Household registration	0.0079	3.15	0.0081	3.21
Region	0.0017	0.69	0.0018	0.71
Healthcare accessibility				
Medical insurance	0.0032	1.25	0.0032	1.26
Reimbursement rate	0.2113	83.93	0.2088	82.92
Distance to health facilities	0.0021	0.84	0.0021	0.83
Unobserved			0.0022	0.86
Total	0.2518	100.00	0.2518	100.00

IOR, relative amount of inequality of opportunity

UC, unobserved circumstances

### Results of robustness tests

The robustness tests of the OLS regression have been mentioned earlier and the results were displayed in Table 2. Table 4 provides the IORs and their decompositions calculated based on the unobserved circumstances extracted from the cluster analysis with 5 and 7 clusters separately. The relative amounts of the IOp and the contributions of all circumstance variables were similar to the results with 6 clusters. Besides, the results of sensitivity analysis were consistent with those obtained by analyzing the samples without missing values (see Additional file 2). However, the coefficient became statistically significant for distance to health facilities.

**Table 4:** Robustness tests based on the cluster analysis with 5 and 7 clusters

Circumstances	5 clusters		7 clusters	
	IOR	Contribution (%)	IOR	Contribution (%)
Socioeconomic status				
Household income	0.0078	3.09	0.0080	3.17
Physical labor	0.0025	1.00	0.0022	0.86
Pension	0.0037	1.47	0.0037	1.46
Education duration	0.0116	4.61	0.0118	4.66
Residence location				
Household registration	0.0081	3.20	0.0082	3.25
Region	0.0018	0.69	0.0017	0.68
Healthcare accessibility				
Medical insurance	0.0032	1.25	0.0031	1.23
Reimbursement rate	0.2093	83.04	0.2078	81.79
Distance to health facilities	0.0021	0.83	0.0021	0.82
Unobserved	0.0021	0.81	0.0053	2.09
Total	0.2520	100.00	0.2541	100.00

IOR, relative amount of inequality of opportunity

UC, unobserved circumstances

## Discussion

To the best of our knowledge, this is the first study to measure the IOp in outpatient expenditure for the elderly with multimorbidity in China. By extracting the heteroscedastic part of the residual for the determining equation of outpatient expenditure between different circumstanced groups, i.e., that stands for the circumstances associated with known circumstances but unobserved, the IOp calculated in this study is closer to the actual value. In addition, the Shapley value decomposition method helped us quantify the contribution of each circumstance factor, providing valuable evidence for improving equity in outpatient service utilization.

The IOp accounted for 25.18% of the total inequality in outpatient expenditure among Chinese elderly aged ≥ 65 years with multimorbidity, similar to 25.9% for middle-aged and older adults aged ≥ 45 years regardless of multimorbidity in 2015 [36]. The decomposition results showed that the contributions of reimbursement rate, education duration, household registration, household income, pension, medical insurance, physical labor, unobserved circumstances, distance to health facilities, and region to the IOp decreased in order. Relying on this ranking, several prioritized and targeted actions can be implemented to reduce the IOp in outpatient expenditure for the multimorbid elderly in China. It should be underlined that pursuing equity in healthcare means moving towards high standards for all, bringing everyone up to the highest common denominator, rather than attempting to lower the previously privileged to the lowest common denominator [47], this is the fundamental principle of this paper to propose recommendations for policy optimization.

### Healthcare accessibility

The magnitude of raising the reimbursement rate of outpatient expenditure cannot be overstated. Previous studies have also shown that increasing the reimbursement rate for outpatient expenditure can significantly promote the utilization of outpatient services by patients with chronic diseases, lower the prevalence and severity of complications, decrease the frequency and length of hospital stays, and alleviate the burden of total medical expenditure [15, 48]. In contrast to the higher reimbursement rate, having medical insurance was significantly associated with lower outpatient expenditure; Reimbursement levels for NRCMS, URBMI/UEBMI, and CMI/FMT increased sequentially, but they contributed far less to the IOp in outpatient expenditure than the reimbursement rate. For the former, the primary reason may be that the multimorbid elderly with medical insurance are more willing to choose hospitalization, or they are typically in favorable social circumstances, whose effect allows them to have better health status [49]. For the latter, the three basic medical insurance schemes in China, NRCMS, URBMI, and UEBMI, provide more generous reimbursement for inpatient care and set high deductibles (the amount paid out-of-pocket for covered healthcare services before the insurer starts to reimburse expenses) for outpatient reimbursement [45, 50]. As an example, if the elderly aged ≥ 65 years in Shanghai enrolled in URBMI went to a secondary health facility seeking care within the city, their outpatient deductible was approximately 45 USD and the reimbursement rate was 55%, while their inpatient deductible was roughly 15 USD and the reimbursement rate was 80% [51]. This disparity may prevent the elderly with multimorbidity from effectively benefiting from the insurance policies. Unlike the ideal reimbursement rates specified by medical insurance schemes, the reimbursement rate for outpatient expenditure employed in this study is the actual value computed based on post hoc data. This re-emphasizes the necessity of refining medical insurance policies to improve the actual reimbursement level of outpatient expenditure for the elderly with multimorbidity. Furthermore, it is important to note that the deductible can also lead to a reverse causal relationship between the actual reimbursement rate and outpatient expenditure. In other words, part of the high contribution of reimbursement rate to outpatient expenditure can be attributed to the presence of deductibles, indicating the imperative of lowering or abolishing the outpatient reimbursement deductible in the three primary medical insurances.

In practice, after the data used in this paper was collected, the Chinese government issued guidelines on improving the outpatient medication security mechanism for urban and rural residents with hypertension and diabetes in 2019 [52]. Many cities have established a series of local reimbursement schemes for chronic diseases designed to lower deductibles and raise rates and ceilings. However, most patients with chronic conditions are unaware of this scheme, and even those who are aware may not be able to take advantage of it due to the limited information regarding its registration process and details [53]. To ensure the multimorbid elderly can be sufficiently covered by the benefits, local governments should organize medical security departments, health institutions, and grassroots medical personnel to carry out policy advocacy employing traditional communication media accessible to the elderly. The results indicate that the association between distance to health facilities and outpatient expenditure was not significant. However, the sensitivity analysis revealed the opposite result. Several factors may explain this finding: the coefficient of distance to health facilities is small and therefore sensitive to minor changes; the increase in sample size reduces the standard error; and potential selection bias. Therefore, more evidence is needed to arrive at a definitive conclusion for multimorbid elderly in China.

### Socioeconomic status

Among the circumstance variables representing socioeconomic status, education duration contributed the most to the IOp in outpatient expenditure for the multimorbid elderly. This may be attributed to the fact that all participants in this study were over the age of retirement. The impact of differences in income and occupation is relatively attenuated, while education has more notable and long-lasting effects on outpatient expenditure. The result that the elderly with multimorbidity who had been educated longer spent more on outpatient visits can be explained by the findings of earlier studies. People with higher educational attainment generally have better health literacy [54], are favored or more proactive in receiving healthcare services [55], and the level of education is considered to affect the individual’s ability to convert information into practical measures and behaviors [56]. Meanwhile, these pieces of evidence reveal possible ways for compensating the elderly with multimorbidity who are in educationally disadvantaged settings. For example, the government can conduct health education campaigns to improve their health literacy, enhance the ability of family physicians to proactively provide healthcare services for them, and implement health promotion initiatives to develop their health management skills. As the elderly with multimorbidity whose household income cannot meet the minimum living standards, medical assistance should be promptly offered. Moreover, patients who previously engaged in physical labor spent more on outpatient services, indicating that the government should pay attention to the health risks and financial burdens arising from poor occupational circumstances. We also found that having a pension did not significantly impact outpatient expenditure, which differs from a recent study on the elderly [57]. One possible explanation is that the stronger association between having a pension and higher health status among the elderly with multimorbidity implies lower medical needs of those with a pension, thereby decreasing outpatient expenditure. This can offset the direct positive effect of pensions in increasing outpatient expenditure, making the coefficient statistically not significant [58].

### Residence location

The household registration system established in 1958 initially aimed to restrict intra-mobility in China, dividing the population into agricultural and nonagricultural sectors, but now leads to severe inequality of opportunity. On the policy side, the social welfare in medical security, endowment insurance, and other policies are incompatible and have an insurmountable divide between urban and rural areas; On the economic side, urban areas possess a disproportionate concentration of high-quality educational and medical resources, and their residents enjoy better occupational circumstances and higher incomes than rural residents [59]. Since the 21st century, the Chinese government has gradually attached importance to the issue and accelerated the pace of urban-rural integrated development [60]. In 2016, all provinces initiated preparations for the merger of NRCMS and URBMI to establish the Urban and Rural Resident Basic Medical Insurance (URRBMI), unifying the fund pooling mechanism and benefits package [61]. However, the URRBMI has not made a considerable dent in the IOp, and the disparity in outpatient expenditure between urban and rural residents appears to be still growing [62]. The possible explanation for this result is that the gap between the rural and urban share of healthcare resources has not shrunk in tandem, with rural residents paying the same amount to purchase medical insurance but struggling to receive healthcare of the same quality locally or incurring more time and transportation costs when traveling to the city, which may limit the reasonable utilization of outpatient healthcare. Furthermore, the difference between URRBMI and UEBMI may also contribute to hindering the reduction of the disparity in outpatient expenditure. Hence, the capacity of medical services in rural areas and the medical security level of URRBMI should be improved as soon as possible, particularly in the treatment and care for the elderly with multimorbidity, which will be the focus of the government’s future health initiatives.

Compared to household registration which has a substantial impact on both economic conditions and healthcare policies, regional disparities in China are mostly attributable to differences in economic development [63]. Pro-rich inequality in health status may be identified as the chief contributing factor to the heavier outpatient expenditure burden for the multimorbid elderly in central and western regions. Although this explanation can also apply to rural elderly, the additional disadvantages of rural areas in social welfare and policies may drive the elderly with multimorbidity to be more inclined to choose treatments with lower costs or even give up treatment, leading to the opposite results in the OLS regression [64]. Improving regional economic equity in a developing China will take a long time. Fortunately, this is not the main determinant of the IOp in outpatient expenditure for the elderly suffering from multimorbidity.

### Limitations

There are still some limitations that need to be acknowledged. Firstly, we utilized the data from a household survey in which chronic disease, outpatient expenditure, and some circumstance information were self-reported, with the possibility of reporting and recall bias. Secondly, because of missing values in the dataset, we had to exclude 31.7% of the cases from the sample, which might result in sample selection biases to a certain degree. Thirdly, we directly took outpatient expenditure as the outcome variable without considering the quality of outpatient services and the additional costs incurred to access them, which may lead to an underestimation of the IOp faced by the multimorbid elderly in China.

## Conclusions

There exists the prominent IOp in outpatient expenditure for the elderly with multimorbidity in China, with the contribution of outpatient reimbursement rate exceeding all other circumstance factors combined, followed by education duration and household registration. The top priority for policy design and perfection is to raise the actual rate of outpatient reimbursement for medications dealing with chronic diseases. Additional crucial strategies encompass promoting health literacy among the multimorbid elderly to facilitate the conversion of their medical needs into effective demands and accelerating the enhancement of medical security and service capacity for chronic diseases in rural areas.

### Electronic supplementary material

Below is the link to the electronic supplementary material.

**Additional file 1**: Results obtained after dealing with the reverse causality caused by large medical expense reimbursement policies

**Additional file 2**: Sensitivity analysis tables

## Data Availability

The dataset analyzed during the current study is available in the Peking University Open Research Data Platform repository, 10.18170/DVN/WBO7LK.

## Abbreviations

IOp: Inequality of opportunity
CLHLS: Chinese Longitudinal Healthy Longevity Study
IOA: Absolute amount of inequality of opportunity
IOR: Relative amount of inequality of opportunity
UC: Unobserved circumstances
NRCMS: New Rural Cooperative Medical Scheme
URBMI: Urban Resident Basic Medical Insurance
UEBMI: Urban Employee Basic Medical Insurance
CMI: Commercial medical insurance
FMT: Free medical treatment
URRBMI: Urban and Rural Resident Basic Medical Insurance
