# Supplementary material for: Inequality of opportunity in outpatient expenditure among the elderly with multimorbidity: evidence from China
Source: Int J Equity Health. 2023 Aug 14;22:153. doi: 10.1186/s12939-023-01953-z (PMC10426157; doi:10.1186/s12939-023-01953-z)
Supplement: Supplementary file 1 — Additional file 1: Results obtained after dealing with the reverse causality caused by large medical expense reimbursement policies [file 12939_2023_1953_MOESM1_ESM.docx]

**Table S1** Circumstance factors influencing outpatient expenditure after dealing with the reverse causality caused by large medical expense reimbursement policies

| Circumstances | Outpatient expenditure (log) | | | |
| --- | --- | --- | --- | --- |
|  | (1) | (2) | (3) | (4) |
| Socioeconomic status | | | | |
| Household income (log) | 0.067*** | 0.067*** | 0.066*** | 0.067*** |
|  | (3.851) | (3.851) | (3.757) | (3.851) |
| Physical labor (ref: no) | 0.288*** | 0.289*** | 0.293*** | 0.287*** |
|  | (4.319) | (4.332) | (4.366) | (4.230) |
| Pension (ref: no) | 0.044 | 0.045 | 0.045 | 0.044 |
|  | (0.688) | (0.707) | (0.708) | (0.688) |
| Education duration | 0.024*** | 0.024*** | 0.025*** | 0.024*** |
|  | (3.666) | (3.662) | (3.730) | (3.666) |
| Residence location | | | | |
| Household registration (ref: urban) | –0.145* | –0.146* | –0.150* | –0.146* |
|  | (–1.684) | (–1.692) | (–1.729) | (–1.690) |
| Region (ref: eastern) | 0.101* | 0.100* | 0.098* | 0.100* |
|  | (1.793) | (1.770) | (1.734) | (1.760) |
| Healthcare accessibility | | | | |
| NRCMS (ref: none) | –0.373*** | –0.373*** | –0.375*** | –0.373*** |
|  | (–3.646) | (–3.643) | (–3.664) | (–3.642) |
| URBMI/UEBMI (ref: none) | –0.296*** | –0.295*** | –0.294*** | –0.296*** |
|  | (–2.886) | (–2.878) | (–2.879) | (–2.879) |
| CMI/FMT (ref: none) | –0.548*** | –0.547*** | –0.547*** | –0.547*** |
|  | (–3.784) | (–3.782) | (–3.800) | (–3.773) |
| Reimbursement rate | 2.129*** | 2.126*** | 2.115*** | 2.128*** |
|  | (27.013) | (26.746) | (26.775) | (26.954) |
| Distance to health facilities | –0.008 | –0.008 | –0.008 | –0.008 |
|  | (–1.618) | (–1.614) | (–1.583) | (–1.616) |
| Unobserved circumstances |  | 0.061 | 0.867 | 0.024 |
|  |  | (0.189) | (0.967) | (0.093) |
| Constant | 5.817*** | 5.816*** | 5.832*** | 5.818*** |
|  | (27.336) | (27.301) | (27.263) | (27.286) |
| Adjusted R^2^ | 0.243 | 0.243 | 0.244 | 0.243 |
| Observations | 3527 | 3527 | 3527 | 3527 |

The unobserved circumstances in columns (2), (3), and (4) were extracted based on the cluster analysis with 6, 5, and 7 clusters, respectively

The t-values calculated based on robust standard errors are reported in the parentheses

*** P < 0.01, ** P < 0.05, * P < 0.1

**Table S2** Decomposition of the IOR in outpatient expenditure after dealing with the reverse causality caused by large medical expense reimbursement policies

| Circumstances | Without UC | | With UC | |
| --- | --- | --- | --- | --- |
|  | IOR | Contribution (%) | IOR | Contribution (%) |
| Socioeconomic status | | | | |
| Household income | 0.0079 | 3.19 | 0.0080 | 3.23 |
| Physical labor | 0.0024 | 0.98 | 0.0025 | 0.99 |
| Pension | 0.0037 | 1.48 | 0.0038 | 1.51 |
| Education duration | 0.0116 | 4.65 | 0.0115 | 4.63 |
| Residence location | | | | |
| Household registration | 0.0080 | 3.20 | 0.0081 | 3.26 |
| Region | 0.0017 | 0.68 | 0.0017 | 0.70 |
| Healthcare accessibility | | | | |
| Medical insurance | 0.0031 | 1.25 | 0.0031 | 1.26 |
| Reimbursement rate | 0.2083 | 83.72 | 0.2057 | 82.68 |
| Distance to health facilities | 0.0021 | 0.85 | 0.0021 | 0.85 |
| Unobserved |  |  | 0.0022 | 0.90 |
| Total | 0.2487 | 100.00 | 0.2488 | 100.00 |

IOR, relative amount of inequality of opportunity

UC, unobserved circumstances

**Table S3** Robustness tests based on the cluster analysis with 5 and 7 clusters after dealing with the reverse causality caused by large medical expense reimbursement policies

| Circumstances | 5 clusters | | 7 clusters | |
| --- | --- | --- | --- | --- |
|  | IOR | Contribution (%) | IOR | Contribution (%) |
| Socioeconomic status | | | | |
| Household income | 0.0078 | 3.15 | 0.0081 | 3.22 |
| Physical labor | 0.0025 | 1.00 | 0.0022 | 0.86 |
| Pension | 0.0037 | 1.49 | 0.0037 | 1.48 |
| Education duration | 0.0117 | 4.68 | 0.0119 | 4.74 |
| Residence location | | | | |
| Household registration | 0.0081 | 3.25 | 0.0083 | 3.30 |
| Region | 0.0017 | 0.69 | 0.0017 | 0.67 |
| Healthcare accessibility | | | | |
| Medical insurance | 0.0031 | 1.25 | 0.0031 | 1.24 |
| Reimbursement rate | 0.2061 | 82.79 | 0.2049 | 81.73 |
| Distance to health facilities | 0.0021 | 0.84 | 0.0021 | 0.83 |
| Unobserved | 0.0022 | 0.87 | 0.0048 | 1.92 |
| Total | 0.2489 | 100.00 | 0.2507 | 100.00 |

IOR, relative amount of inequality of opportunity

UC, unobserved circumstances
