# Supplementary material for: Inequality of opportunity in outpatient expenditure among the elderly with multimorbidity: evidence from China
Source: Int J Equity Health. 2023 Aug 14;22:153. doi: 10.1186/s12939-023-01953-z (PMC10426157; doi:10.1186/s12939-023-01953-z)
Supplement: Supplementary file 2 — Additional file 2: Sensitivity analysis tables [file 12939_2023_1953_MOESM2_ESM.docx]

**Table** **S1** Circumstance factors influencing outpatient expenditure based on the imputed datasets (imputations = 20)

| Circumstances | Outpatient expenditure (log) | |
| --- | --- | --- |
|  | Without UC | With UC |
| Socioeconomic status |  |  |
| Household income (log) | 0.055*** | 0.055*** |
|  | (4.054) | (4.061) |
| Physical labor (ref: no) | 0.249*** | 0.247*** |
|  | (4.460) | (4.416) |
| Pension (ref: no) | 0.035 | 0.036 |
|  | (0.676) | (0.691) |
| Education duration | 0.022*** | 0.023*** |
|  | (3.998) | (4.035) |
| Residence location |  |  |
| Household registration (ref: urban) | –0.125* | –0.127* |
|  | (–1.828) | (–1.851) |
| Region (ref: eastern) | 0.098** | 0.096** |
|  | (2.149) | (2.081) |
| Healthcare accessibility |  |  |
| NRCMS (ref: none) | –0.291*** | –0.290*** |
|  | (–3.667) | (–3.658) |
| URBMI/UEBMI (ref: none) | –0.186** | –0.183** |
|  | (–2.240) | (–2.212) |
| CMI/FMT (ref: none) | –0.430*** | –0.425*** |
|  | (–3.606) | (–3.575) |
| Reimbursement rate | 2.148*** | 2.150*** |
|  | (33.360) | (33.297) |
| Distance to health facilities | –0.010*** | –0.010*** |
|  | (–3.757) | (–3.758) |
| Unobserved circumstances |  | 0.174 |
|  |  | (0.593) |
| Constant | 5.831*** | 5.831*** |
|  | (35.279) | (35.288) |
| Adjusted R^2^ | 0.238 | 0.239 |
| Observations | 5163 | 5163 |

The unobserved circumstances were extracted based on the cluster analysis with 10 clusters The t-values calculated based on robust standard errors are reported in the parentheses

UC, unobserved circumstances

*** *P* < 0.01, ** *P* < 0.05, * *P* < 0.1

**Table** **S2** Decomposition of the IOR in outpatient expenditure based on the imputed datasets (imputations = 20)

| Circumstances | Without UC | | With UC | |
| --- | --- | --- | --- | --- |
|  | IOR | Contribution (%) | IOR | Contribution (%) |
| Socioeconomic status | | | | |
| Household income | 0.0069 | 2.83 | 0.0070 | 2.84 |
| Physical labor | 0.0020 | 0.80 | 0.0019 | 0.77 |
| Pension | 0.0037 | 1.50 | 0.0037 | 1.50 |
| Education duration | 0.0112 | 4.56 | 0.0113 | 4.60 |
| Residence location | | | | |
| Household registration | 0.0069 | 2.83 | 0.0070 | 2.85 |
| Region | 0.0013 | 0.55 | 0.0013 | 0.53 |
| Healthcare accessibility | | | | |
| Medical insurance | 0.0029 | 1.20 | 0.0029 | 1.18 |
| Reimbursement rate | 0.2060 | 84.21 | 0.2066 | 84.22 |
| Distance to health facilities | 0.0037 | 1.50 | 0.0037 | 1.50 |
| Unobserved |  |  | 0.0010 | 0.39 |
| Total | 0.2446 | 100.00 | 0.2453 | 100.00 |

The unobserved circumstances were extracted based on the cluster analysis with 10 clusters

IOR, relative amount of inequality of opportunity

UC, unobserved circumstances
